# Exploring Patient Satisfaction and Other Outcome Measures With Pain Relief in Spinal Cord Stimulation: A Single-Site, Cohort Audit

**DOI:** 10.7759/cureus.51339

**Published:** 2023-12-30

**Authors:** Mohammad Saleki, Mazen Khabbass, Beatrice Bretherton, Ganesan Baranidharan

**Affiliations:** 1 School of Medicine, University of Leeds, Leeds, GBR; 2 Pain Management, Leeds Teaching Hospitals NHS Trust, Leeds, GBR

**Keywords:** numerical rating scale, audit, satisfaction, pain, spinal cord stimulation (scs)

## Abstract

Context

Spinal cord stimulation (SCS) is an approved treatment for chronic pain of neuropathic origin. Initial research suggests a close relationship between pain relief and patient satisfaction with SCS.

Objectives

To see whether similar patterns were observed in our center and to identify areas of improvement, this single-site, cohort audit explored the association between pain relief and satisfaction as well as specific factors that influence satisfaction at follow-up in patients with fully implanted SCS.

Methods

Age, gender, pain condition, SCS system, average pain (numerical rating scale, NRS), and worst pain (NRS) scores at baseline and the most recent follow-up visit were collected. Percentage change in average pain was calculated, and the patients were allocated to one of three pain improvement groups: <30%, 30%-50%, or >50%. A telephone survey explored patient outcomes including patient satisfaction, sleep, fatigue, quality of life (QoL), walking ability, and medication use. Chi-square tests of independence were performed.

Results

The final sample comprised 87 patients (<30%: n = 26; 30%-50%: n = 29; >50%: n = 32). The pain improvement group was significantly associated with satisfaction (p = 0.010): all patients in the >50% improvement group reported being either very satisfied or somewhat satisfied with SCS. The pain improvement group was also significantly associated with change in sleep (p < 0.001), fatigue (p = 0.001), QoL (p = 0.003), and opioid consumption (p = 0.010). Improvements were most frequently reported in the >50% improvement group.

Conclusion

Findings point to an association between pain relief and patient satisfaction with SCS. Other factors, including sleep, fatigue, QoL, and opioid consumption, may influence this association and deserve further exploration.

## Introduction

Neuropathic pain affects 7%-8% of adults [1] and can be caused by a lesion or disease in the somatosensory nervous system [2]. It is challenging to treat; less than 50% of patients with neuropathic pain respond to pharmacological treatment [3]. As medication is associated with side effects, there has been a demand for alternative therapies that are safer and more effective. In the United Kingdom, spinal cord stimulation (SCS) is a National Institute for Health and Care Excellence (NICE)-approved treatment for adults who have had chronic pain of neuropathic origin for more than six months despite conservative treatment [4]. Although findings from randomized controlled trials show evidence of efficacy and safety of SCS [5-13], this has been recently challenged [14,15], calling into question the benefits of SCS and prompting multiple institutions to further investigate.

SCS efficacy is typically ascertained by reductions in pain intensity. However, given the recent controversy of outcomes of SCS which are contrary to the established evidence of the benefits of SCS, questions can be raised about how SCS outcomes are measured, e.g., numerical rating scale (NRS) for pain intensity. Additionally, due to inter-person variability in the interpretation of pain, it can be challenging to determine what a change in pain intensity means. For example, despite reporting a one-point reduction in NRS pain score from 8 to 7 at 12 months following SCS, a patient may be satisfied with the pain relief. In contrast, a patient who reports a reduction of three points from 8 to 5 following SCS may not be as satisfied with their treatment as the former patient. Indeed, it is not unusual for some patients to report high satisfaction with pain therapy while also reporting modest reductions in pain intensity [16,17]. It is therefore crucial to consider patient satisfaction with SCS treatment when assessing the impacts of SCS in patients. Indeed, in a retrospective evaluation of outcomes with SCS, the intensity of back pain at 24 months was negatively correlated with patient satisfaction (measured on a five-point Likert-type scale) [18]. Prospective open-label and retrospective trials suggest that SCS is associated with high rates of satisfaction [19-22]. Furthermore, randomized controlled trials have shown that patient satisfaction may be influenced by treatment modality [5,13,23] and may be predicted by several factors, e.g., evening pain intensity and an interaction between evening pain intensity and walking tolerance time [24]. It is therefore imperative to investigate whether we can further improve the outcomes of SCS by breaking down the most important factors to improving patient satisfaction with SCS.

To see whether data from our center reached a similar outcome and to identify areas of improvement to encourage better outcomes for patients, we conducted this single-site, retrospective cohort audit to explore the association between pain relief and satisfaction in patients with fully implanted SCS. This audit was exploratory (i.e., not hypothesis-driven). The findings of this audit also contribute to the ongoing assessment of outcome measures for SCS and the exploration of dependable factors that impact patient-reported pain while also providing general outcome data for SCS scrutinization.

## Materials and methods

This was a single-site, retrospective cohort audit that explored pain relief and satisfaction in patients with 10 kHz or BurstDR SCS. This was a service improvement project done as a part of the Extended Student-Led Research or Evaluation (ESREP) program. The project went through the university vetting on audit versus research and was deemed an audit/service evaluation. It was performed within the NHS audit governance framework after registration in our NHS Trust audit database (reference: 8639). As there was no direct patient contact, these were short over-the-phone surveys where patients had the option of opting out, and our audit retrospectively collected data for comparison; therefore, our university board review deemed ethical approval was not required.

Patients and data collection

Patients aged ≥18 years who had fully implanted 10 kHz or BurstDR SCS between February 2013 and March 2020 were identified from hospital paper and electronic records. These dates were used due to ease of access within the department. As these two waveforms were predominantly used at our establishment during the period, we focused on these waveforms. Scores for average pain and worst pain (ascertained via NRS) during standard clinical practice visits at baseline and follow-up were collected. Our standard clinical practice visits were as follows. Postoperatively, patients attended a two-week wound check. They would then attend the department at six weeks, three months, and six months following the surgery for outcomes to be monitored. Patients who required re-programming for greater pain relief would also be offered re-programming at these appointments. After one year following surgery, patients attended a follow-up and discharge appointment. If, within this time, patients required troubleshooting (e.g., re-programming for greater pain coverage), they would be seen in the department during further appointments. The date of the baseline and follow-up visits were recorded, together with age, gender, pain condition, and SCS system. Percentage change in average pain between baseline NRS and the most recent follow-up visit NRS was calculated.

Pain response has previously been defined by a reduction in pain score of ≥30% [25], ≥40%, [26], ≥50% [7,8,11,27], and ≥80% [11]. This, combined with the possibility that some patients may report a small change in pain relief while being satisfied with SCS (evidenced by continued use of SCS), resulted in the creation of the following groups: Group 1: <30% reduction in average pain NRS; Group 2: 30%-50% reduction in average pain NRS; and Group 3: >50% reduction in average pain NRS.

All patients who had fully implanted 10 kHz or Burst SCS and NRS scores for average pain at baseline and the most recent follow-up visit were anonymously entered into a spreadsheet and allocated an audit ID number. To ensure appropriate representation of each group in the final sample as well as account for patients who may not have been contactable and/or declined the telephone contact, 50 patients from each group were randomly selected using the Google random number generator (n = 150 patients in total).

Telephone contact included a combination of closed-ended and free text questions about patient outcomes of their SCS (see Appendix). A script for the telephone contact was created to ensure the data collected were reproducible between the two team members undertaking the telephone contact. Responses were recorded directly in an Excel document, with free text answers recorded verbatim.

Responses were collected on satisfaction, sleep, fatigue, quality of life (QoL), sexual function, general activities, walking ability, work, socializing, medication consumption, unexpected benefits, negative effects, and SCS recommendation. Satisfaction was measured using a five-point Likert scale, and data for sleep, fatigue, QoL, sexual function, general activities, walking ability, work, and socializing were collected using the Global Impression of Change Score. Medication use was coded as either stopped, decreased, stayed the same, or increased.

Data analysis

Age differences, the time between implant and follow-up, and the time between implant and telephone contact between the three groups were explored by one-way ANOVAs (or Kruskal-Wallis tests for non-normally distributed data) and Bonferroni pairwise comparisons. Normality was ascertained by the Shapiro-Wilk test.

The Bonferroni correction was used for multiple pairwise comparisons when one-way ANOVAs or Kruskal-Wallis tests were statistically significant. No further adjustments for multiple comparisons were made due to the exploratory nature of this audit. However, we have acknowledged the limitation of this statistical approach in the discussion section. Chi-square tests of independence explored associations between the three groups and various factors, such as gender, SCS system, satisfaction, sleep, fatigue, QoL, sexual function, general activities, walking ability, work, socializing, medication consumption, unexpected benefits, negative effects, and SCS recommendation.

The alpha level was set to 0.05, and all statistical tests were two-tailed. All statistical analyses were conducted in Statistical Package for the Social Sciences (SPSS) version 25 (IBM Corp., Armonk, NY). As the telephone contact included free text answers, these qualitative data were combined with the statistical analyses to provide further details about patient perspectives regarding SCS.

## Results

Sample characteristics

**Figure 1:**
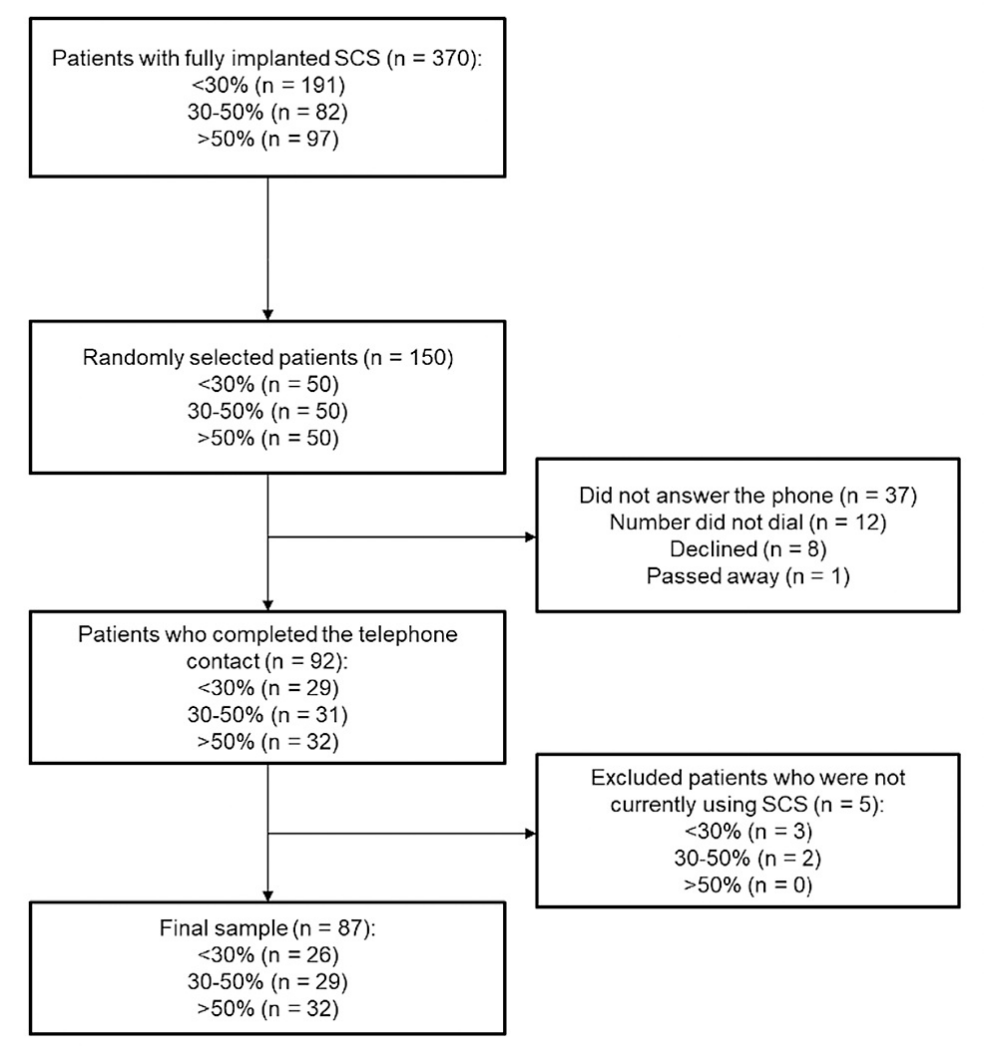
Summary of patient flow SCS: Spinal cord stimulation.

Three hundred and seventy patients who had fully implanted 10 kHz or Burst SCS and average pain NRS scores at baseline and the most recent follow-up visit were identified. Percentage change in average pain between baseline and the most recent follow-up visit was calculated, and the patients were allocated to one of the three groups: <30% (n = 191), 30%-50% (n = 82), or >50% (n = 97). One hundred and fifty patients were then randomly selected to be contacted (50 patients in each group). Thirty-seven patients did not answer the phone, 12 numbers did not dial, eight declined, and one patient died. For the 37 patients who did not answer the phone, each patient was called twice on two separate occasions, after which no further phone calls were made to avoid spam calls. This resulted in 92 patients (<30%: n = 29; 30%-50%: n = 31; >50%: n = 32). Five patients were not using their SCS at the time of the telephone contact and were excluded from all subsequent analyses. This resulted in a final sample of 87 patients (<30%: n = 26; 30%-50%: n = 29; >50%: n = 32). Figure 1 summarizes the patient flow, and Table 1 summarizes the baseline characteristics of the final sample.

**Table 1:** Summary of baseline characteristics for the final sample Data are presented raw for gender, system, and diagnosis, median (interquartile range [IQR]) for baseline average pain, worst pain, the time between the implant and most recent follow-up visit, the time between the implant and telephone contact, and mean (standard deviation) for age. SCS: Spinal cord stimulation; FBSS: Failed back surgery syndrome; CRPS: Complex regional pain syndrome; NRS: Numerical rating scale.

		Final sample (n = 87)
			Gender	Males	36
Females	51		
System	10 kHz SCS	44
	Burst SCS	43
Diagnosis	FBSS	50
	CRPS	9
	Visceral	9
	Peripheral mononeuropathy	8
	Pelvic/perineal pain	5
	Other (post-amputation, brachial avulsion, spinal cord injury, and critical limb ischemia)	6
Baseline	Average pain (NRS)	7.00 (1.00)
	Worst pain (NRS)	9.00 (2.00)
Age (years)		54.48 (12.13)
Time between the implant and	Most recent follow-up visit (months)	12.95 (25.35)
	Telephone contact (months)	32.48 (29.13)

Gender and system were not significantly associated with group (gender: p = 0.503; SCS system: p = 0.566). Also, age (p = 0.794), time between implant and follow-up (p = 0.334), and time between implant and telephone contact (p = 0.167) did not significantly differ between the three groups (Table 2).

**Table 2:** Summary of baseline characteristics according to the group Data are presented raw for gender and system, mean (standard deviation) for age, and median (IQR) for the time between the implant and the most recent follow-up and the time between the implant and telephone contact. SCS: Spinal cord stimulation.

		Group		
		<30% (n = 26)	30%-50% (n = 29)	>50% (n = 32)
Gender	Males	13	10	13
	Females	13	19	19
System	10 kHz SCS	15	15	14
	Burst SCS	11	14	18
Age (years)		55.15 (10.72)	55.17 (13.77)	53.31 (11.91)
Time between the implant and the most recent follow-up (months)		12.45 (29.31)	17.23 (19.20)	11.77 (21.67)
Time between the implant and telephone contact (months)		30.15 (34.25)	36.16 (28.50)	30.64 (23.32)

Satisfaction with SCS

Forty-eight patients (55%) were very satisfied with SCS, and 33 patients (40%) were somewhat satisfied, totaling 81 patients (93%) who were satisfied with SCS. Satisfaction was significantly associated with groups (p = 0.010): all patients (100%) in the >50% group reported satisfaction with SCS; 88% and 89% of patients in the <30% and 30%-50% groups, respectively, reported satisfaction with SCS; 8% and 3% of patients in the <30% and 30%-50% groups, respectively, reported dissatisfaction with SCS; and 4% and 7% of patients in the <30% and 30%-50% groups, respectively, reported being neither satisfied nor dissatisfied with SCS.

Positive effects

Changes in sleep, fatigue, QoL, sexual function, general activities, walking ability, work, and socializing were ranked by patients on a seven-item scale. To aid with analysis, this scale was condensed into three categories: "improved," "no change," and "worse." Regardless of group, most patients reported improvements in sleep (59%), fatigue (52%), QoL (85%), general activities (78%), walking ability (67%), work (61%), and socializing (76%). For sexual function, most patients reported no change (68%). Group was significantly associated with the change in sleep (p < 0.001), fatigue (p = 0.001), and QoL (p = 0.003). Improvements in sleep, fatigue, and QoL were most frequently reported in patients with >50% reduction in average pain. There were no statistically significant associations between group and change in sexual function, general activities, walking ability, work, and socializing (p > 0.050).

Using the seven-item scale, similar patterns emerged for sleep (p = 0.011), fatigue (p = 0.012), and QoL (p = 0.001). There were no statistically significant associations between group and change in sexual function, general activities, walking ability, work, and socializing (p > 0.050).

Medication use

Three patients were not using opioids at the time of receiving fully implanted SCS. Opioid consumption stopped in 25% of patients, decreased in 38%, stayed the same in 33%, and increased in 4%. Opioid consumption was significantly associated with group (p = 0.010). A greater percentage of patients had stopped opioid medication, and a smaller percentage had no change in opioid medication in the >50% group compared to the <30% and 30%-50% groups. For some patients, the decrease in opioid consumption was due to a dislike of taking the medication and the associated side effects. In addition, increasing safety while driving was an additional important factor in reducing opioid medication. For those who reported medication increases, the reasons included the emergence of new pain.

Twenty patients were not using neuropathic pain medication at the time of receiving SCS. Neuropathic pain medication decreased in 34% of patients, stayed the same in 16%, and increased in 49%. There was no statistically significant association between the change in neuropathic pain medication and the group (p > 0.05).

Unexpected benefits of SCS

Thirty-four patients (of 87, 39%) reported unexpected benefits. The percentage of patients who reported unexpected benefits was comparable between the three groups (<30%: 35%; 30%-50%: 38%; >50%: 44%, p > 0.05). When prompted for further details, some commented they had not anticipated their QoL to have improved to the extent it had. Some patients even noted that the improvements in mental health, mood, and personality had been unexpected. Enhanced physical activity, such as being able to exercise, lift weights, walk, cycle, and do more gardening were further unanticipated benefits. Some also reported they could drive, did not require walking sticks or a mobility scooter, could stand up straight, had better balance, and were more flexible due to their SCS. Regarding the magnitude of pain relief, some patients were "pleasantly surprised by the amount of pain reduction," with some acknowledging that their night-time pain had completely gone, their legs did not burn when moving, and they could get out of bed in the morning, thanks to the SCS. Others also acknowledged that the SCS helped to control spasms and, as a result, reduced the associated apprehension. Improvements in sleep were unexpected benefits for some patients, along with being able to return to work, stopping medication, and not vomiting as often.

Negative effects of SCS

Most patients (regardless of group) did not experience increased pain (68 of 87, 78%). However, some patients reported that pain relief was variable and could be less than the pain relief they experienced compared to when the SCS system was first implanted. Another patient noted the SCS trial provided greater pain relief compared to their fully implanted system. Although 84% of patients reported they did not experience unexpected numbness, 14% noted there were occasions when they did feel numbness. When prompted for further details, the numbness occurred at the top of the spine and in the fingers. The prevalence of infection and the anchor site pain were low (7% and 28%, respectively). Implantable pulse generator (IPG) site pain was reported by 22% of patients with comments highlighting the occurrence of painful or uncomfortable sensations at the IPG site when people bumped into them, when they changed position (e.g., from standing to sitting), or when it caught on clothing or other items. For other patients, burning sensations occurred at the IPG site when recharging due to the battery heating up. Recharging was perceived as a burden by 23%. Some patients noted this was because the battery required longer amounts of time to recharge. Other unexpected negative effects included experiencing electrical shocks, burning and tingling sensations in the legs, headache (although this was dependent on the program used), stiff neck, tiredness, and challenges when traveling (particularly via airports). Some patients suggested the design could be improved by reducing the weight and size of the implant. There were no statistically significant associations between these unexpected negative effects and group (p > 0.050).

Would patients recommend SCS?

About 94% of patients would recommend SCS, 5% were unsure, and 1% would not recommend it. Although the recommendation was not significantly associated with a group (p = 0.298), all patients in the >50% group said they would recommend SCS. Some patients highlighted that SCS had changed their lives, was the best thing they had done, and that they had recommended it to friends who had received fully implanted SCS. Other patients maintained that although comorbidities had reduced the benefits associated with SCS, overall, they were happy that they underwent the surgery.

## Discussion

This single-center, retrospective cohort audit explored the association between pain relief (in different regions including the back) and patient satisfaction following SCS. Findings revealed a significant association between pain relief (measured as average pain NRS) and patient satisfaction (measured on a five-point Likert-type scale). In the group of patients with >50% reduction in average pain, all reported being either very or somewhat satisfied with SCS, whereas all patients who were very dissatisfied were in the <30% group. Findings also showed that the group was significantly associated with changes in sleep, fatigue, QoL, and opioid consumption, where improvements were most frequently reported in patients in the >50% group. Taken together, findings suggest that outcomes in addition to pain relief may be important in influencing patient satisfaction with SCS.

Patient satisfaction with SCS

Satisfaction and pain severity are correlated in pain patients [17,18] with pain severity potentially predicting satisfaction with SCS [24]. Findings from the present audit revealed a statistically significant association between pain relief and patient satisfaction with SCS when average pain was quantified by NRS and satisfaction on a five-point Likert-type scale. About 81% of patients reporting >50% reduction in average pain were very satisfied with their outcomes compared to 41% and 38% of patients reporting 30%-50% and <30% reduction in average pain, respectively. These findings suggest that pain relief with SCS may be associated with patient satisfaction in our center, which will be included in our standard clinical practice going forward. The findings are also consistent with those used as a benchmark for the present audit [18], although there are key methodological differences (e.g., back pain versus pain in different regions including the back and visual analog scale [VAS] versus NRS).

Due to the small number of patients who reported being very dissatisfied, somewhat dissatisfied, or neither satisfied nor dissatisfied with their SCS (n = 6), it was not possible to statistically test the differences in outcomes between the satisfaction levels. We therefore explored the differences in the outcomes between the three groups. Findings highlighted the potential of SCS to have positive effects in other domains when greater pain relief was reported, including sleep, fatigue, and QoL. Improvements in sleep and QoL following SCS have been evidenced in previous research [26]. These findings therefore provide evidence of the significant positive effects of SCS on patient outcomes, challenging recent studies [14,15] and underscoring the overall positive impact of SCS on patients' well-being.

Analgesia and pain relief

Consistent with previous research showing an association between SCS and decreases in opioid analgesia [7,19,21,28], 25% of patients stopped opioids following SCS, and 38% decreased their dose. Although the decrease in neuropathic pain medication appeared greater in the >50% group (48% of patients) compared to the <30% group (17% of patients), there was no statistically significant association between the group and the change in neuropathic pain medication. Unexpectedly, findings showed that 49% of all patients increased their need for neuropathic pain medications, with 67% of the <30% group needing to do so. While previous treatment with SCS was not ascertained for this audit, we realize this finding is in contrast to previous research, which showed that high-dose SCS in patients with FBSS was associated with a decrease in neuropathic pain agents in SCS-naive patients but not in rescue patients [29]. Additionally, combination therapy of neuropathic pain medication (namely duloxetine) with SCS yielded more positive outcomes, and patients taking duloxetine would be more willing to have SCS again [30]. Taking neuropathic pain medication may have, therefore, improved SCS outcomes, and 49% of patients in our audit who increased their neuropathic pain medication may have improved their SCS outcomes.

Limitations and future directions

This retrospective cohort audit, held in a single center with a participation rate of 50%-60%, likely carried biases that a prospective trial could correct. It focused on 10 kHz and Burst SCS waveforms, prominent in our center at the time, but many other waveforms warrant exploration in future studies. The absence of documented comorbidities in patient groups might have influenced their responses. Alternate patient cohort methods could have reduced variability, like reaching out to patients with a single waveform over a specific duration. Despite similar timeframes between implant, follow-up, and telephone contact across three groups, notable variability, seen in large standard deviations, might have affected the findings. Nevertheless, this approach aimed to kickstart the evidence base for pinpointing service improvements and guiding future study designs. In addition, as this was an exploratory audit and not hypothesis-driven, the approach adopted here aims to help set the scene of how satisfaction, and other variables, relate to pain relief with SCS. Hence, it is important to note that our study did not differentiate between patients using neuropathic pain medications and those who were not, potentially confounding the results. We acknowledged that the statistical approach adopted could be improved in future research by more robustly analyzing the data (e.g., models that control for confounding variables and multiple comparisons).

The tools employed to measure these factors should be considered thoughtfully, prioritizing validated methods. For instance, our standard practice involves using the EQ-5D questionnaire during baseline and follow-up visits to gauge changes in health-related QoL. However, during telephone contacts, we assessed QoL using a seven-point Likert-type scale. Consequently, comparing these two measures became impractical. Moreover, while exploring methods to quantify patient satisfaction, we found that previous research had employed a wide range of different measurement approaches, for example, a binary response (yes/no) [16] and three-point [22], five-point [13,19,21], six-point [17], and seven-point [24] scales. While the five-point scale used in this audit provides some distinction between very dissatisfied/somewhat dissatisfied and very satisfied/somewhat satisfied, the seven-point scale (as used in [24]) would provide greater granularity. Although we used a seven-point scale to explore the secondary outcomes, this provided too much granularity, leading us to compress into three groups for the analysis. While we thought this seven-item scale would generate greater insight into the secondary outcomes, on reflection, this was a limitation that should be carefully considered in future research. Future research should also include factors that patients perceive to be important when considering their satisfaction with SCS. This would require patient and public involvement and engagement to understand the factors that contribute to their satisfaction with SCS. In addition, as this was an exploratory audit and not hypothesis-driven, the approach adopted here aims to help set the scene of how satisfaction and other variables relate to pain relief with SCS. We acknowledged that the statistical approach adopted could be improved in future research by more robustly analyzing the data (e.g., models that control for confounding variables and multiple comparisons).

## Conclusions

This audit revealed a statistically significant association between pain relief and patient satisfaction. Due to the high percentage of patients who were either satisfied or very satisfied with SCS (93%), it was not possible to statistically test the differences between satisfaction levels. However, positive effects of SCS were observed in other domains (sleep, fatigue, QoL, and opioid consumption) when greater pain relief was reported. Sleep, fatigue, QoL, and opioid consumption may be four key outcome measures for evaluating patient satisfaction with SCS. This is in line with the anecdotal conversations with patients who felt little benefit in documenting pain scores; instead, they discussed how their lives had changed post-SCS. With large, prospective research examining the role additional outcomes play in modulating the relationship between pain relief and patient satisfaction with SCS, it will be possible to better prioritize outcome measures to judge the success of SCS.

## Appendices

**Table 3:** Telephone survey questions PGIC scoring is as follows: 1 = very much improved, 2 = much improved, 3 = minimally improved, 4 = no change, 5 = worse, 6 = much worse, and 7 = very much worse. SCS: Spinal cord stimulation; PGIC: Patient Global Impression of Change.

Questions	Answer options
1. Are you still using your SCS device?	Yes/No
a. If no, reason:	i. Recharge burden
	ii. My pain is better now
	iii. Caused more pain
	iv. Does not work anymore
	v. Other – free text
2. Overall, how satisfied are you with your SCS implantation?	Very satisfied
	Somewhat satisfied
	Neither satisfied nor dissatisfied
	Somewhat dissatisfied
	Very dissatisfied
3. Using the PGIC, how much do you feel your function improved in the following?	
a. General activities (e.g., showering, dressing, cooking)	1-7
b. Walking ability (e.g., walking around shops)	1-7
c. Ability to function at work	1-7
d. Ability to socialize with friends and family	1-7
e. Sleep	1-7
f. Fatigue	1-7
g. Sexual function	1-7
h. Other	Free text
4. How would you rate your quality of life now using PGIC scoring?	1-7
5. Side effects	
a. Do you use a rechargeable battery? If yes, ask below.	Yes/No
i. How often do you recharge your device?	More than weekly
	Weekly
	Fortnightly
	Monthly
	Other: free text
ii. Do you find recharging it a burden?	Yes/No
b. Have you experienced any of the following negative effects?	
i. Increased pain	Yes/No
ii. Unexpected numbness	Yes/No
iii. Infection	Yes/No
iv. Anchor site pain	Yes/No
v. Implantation site pain	Yes/No
vi. Recharge burden	Yes/No
vii. Other	Free text
6. Have you experienced any unexpected benefits?	Yes/No
7. Has your use of opioids stopped, decreased, stayed the same, or increased?	Stopped
	Decreased
	Stayed the same
	Increased
8. Has your use of anti-nerve painkillers stopped, decreased, stayed the same, or increased?	Stopped
	Decreased
	Stayed the same
	Increased
9. Would you recommend SCS to a friend?	Yes/No
